# Interventionalists’ perceptions on a culture of radiation protection

**DOI:** 10.4102/sajr.v22i1.1285

**Published:** 2018-03-19

**Authors:** André Rose, Kerry E. Uebel, William I. Rae

**Affiliations:** 1 Department of Community Health, University of the Free State, South Africa; 2 Department of Internal Medicine, University of the Free State, South Africa; 3 Department of Medical Physics, University of the Free State, South Africa

## Abstract

**Background:**

Occupational exposure to ionising radiation poses potential health risks to radiation workers unless adequate protection is in place. The catheterisation laboratory is a highly contextualised workplace with a distinctive organisational and workplace culture.

**Objective:**

This study was conducted to understand the culture of radiation protection (CRP).

**Methods:**

This study was a qualitative study and data were collected through 30 in-depth and 6 group interviews with 54 purposively selected South African interventionalists (interventional radiologists and cardiologists). The participants included a diversity of interventionalists who varied in sex, geographic location and years of experience with fluoroscopy. The transcribed data were analysed thematically using a deductive and inductive approach.

**Results:**

‘Culture of radiation protection’ emerged as a complex theme that intersected with other themes: ‘knowledge and awareness of radiation’, ‘radiation safety practice’, ‘personal protective equipment (PPE) utilisation’ and ‘education and training’.

**Conclusion:**

Establishing and sustaining a CRP provides an opportunity to mitigate the potentially detrimental health effects of occupational radiation exposure. Education and training are pivotal to establishing a CRP. The time to establish a culture of radiation in the catheterisation laboratory is now.

## Introduction

Ionising radiation is a modality that is neither seen nor heard and is intangible. It is a health hazard for patients and radiation healthcare workers (HCWs). Interventionalists are doctors who perform fluoroscopically guided procedures for diagnosing or treating patients. They include interventional cardiologists and interventional radiologists. Vascular surgeons, urologists, orthopaedic surgeons and anaesthesiologists may also perform fluoroscopic procedures as part of their practice. Interventionalists are not always aware of the detrimental long-term effects that radiation exposure may have on their health and may be negligent in implementing and consistently applying radiation safety practices.

The increasing use of fluoroscopy for diagnostic, therapeutic and interventional procedures necessitates greater vigilance in the use of this modality within the workplace. The increasing trend in ionising radiation utilisation in medical procedures has increased diagnostic exposure from X-rays, computed tomography (CT) scans, mammography and nuclear medicine scans.^1^ Therapeutic use includes angioplasty, radiation therapy for tumours and therapeutic use of radioisotopes. Interventional practices include fluoroscopic procedures like radio-ablation, pacemaker insertion and embolisation.^1^ There has been an increase in the duration and complexity of these procedures, resulting in greater cumulative occupational exposure to operators.^2^

The most at risk professions include interventional radiologists and cardiologists.^3^ Operators performing fluoroscopic work have an increased occupational risk of developing ionising radiation-induced health detriments.^4^ The effects of ionising radiation exposure may be stochastic or deterministic.^5^ Stochastic effects have no threshold limit and occur randomly; for example, genetic aberrations and cancers. Deterministic effects occur when an exposure threshold is exceeded and the outcomes include skin changes; they may also occur in patients who are overexposed.^6^ Protection of radiation HCWs in the workplace is essential because they may experience effects such as cataracts.^7^

Protection from ionising radiation in the workplace hinges on providing an enabling environment for radiation safety, application of the as low as reasonably achievable (ALARA) principle, monitoring of the radiation dose received (dosimetry), complying with international occupational dose limits and regular and consistent use of personal protective equipment (PPE). All of these aid in creating a culture of radiation protection (CRP) in the workplace.^8,9^ This culture is underpinned by a set of norms and values that shape how it is developed, implemented and maintained.^10^ It is thus vital that a CRP should be developed, implemented and sustained to protect radiation healthcare professionals.^11^

A CRP is imperative for protecting HCWs from the effects of radiation. Such a culture includes compliance with the radiation regulations, use of PPE, compliance by all of the radiation team and adequate monitoring and evaluation.^2^ The genesis of such a culture is sparked and sustained by adequate education and training.^12^

Personal protective equipment is used to reduce the exposure to radiation for HCWs, thereby minimising the risk of radiation health effects.^13^ The range of PPE includes ceiling suspended screens, lead aprons, lead caps, thyroid guards and lead glasses or visors.^14^ The effectiveness of PPE depends on its consistent use. Consistency of use depends on availability, fit of the PPE and the comfort when performing procedures.^15^ A recent study has shown that when workers have access to PPE, it is more likely to increase their utilisation of it.^16^ In a survey on attitudes of PPE usage, it was found that certain devices such as lead aprons and thyroid shields were widely used and considered ‘standard practice’, while other devices that protected more radiosensitive organs such as the lenses of the eyes were used inconsistently.^17^

The behaviour in an environment like the catheterisation laboratory depends on the interplay between the attitude of individuals, the comportment of professionals and the engagement between administrative and managerial staff.^18^ Organisational culture in a health facility is shaped by four key factors: responsiveness, team hierarchy, care philosophy and communication.^19^ These dynamic interactions are a conduit to the barriers and facilitators which shape a CRP.

Formal training in radiation safety reduces the dose to patients and HCWs in the catheterisation laboratory.^12^ Formalised training and continued education on radiation safety help establish and sustain a CRP in the workplace. Radiation safety training and education needs to form part of the formalised curriculum of radiation HCWs in order for it to be taken seriously.^20^ Continued medical education (CME) can help consolidate and sustain the training and preservation of a CRP to ensure a safer workplace.^21^ This CME programme should be consistent and reinforced regularly to maintain its effectiveness.^12^

The aim of this article is to present the perceptions of interventionalists on what constitutes a CRP. The findings reflect on the challenges for developing a CRP in the workplace, how to establish such a culture and how to continue to foster it. We reflect on how these principles can be universally applied.

## Methods

### Study design

This study had a qualitative design which formed part of a larger multiple methods study which is described elsewhere.^22^

### Participant selection

The participants were all interventionalists and included adult cardiologists, paediatric cardiologists and interventional radiologists with different levels of expertise. They included specialists in training, junior and senior specialists and heads of departments. We purposefully included a diversity of participants to reflect the gender, number of years worked, different professional categories, sectors worked in, levels of training and cities worked in South Africa – a total of 54 participants (see Table 1).

**TABLE 1:** The characteristics of 54 participants in six group interviews and 30 in-depth interviews.

Variable	In training	Junior^†^	Mid career^‡^	Senior^§^	HOD
**Sex**					
Male	12	1	6	10	4
Female	9	3	6	1	2
**Occupation**					
Radiologist	-	1	7	5	2
Adult cardiologist	-	1	2	5	2
Paediatric cardiologist	-	2	3	1	2
Paediatric fellow	2	-	-	-	-
Cardiology fellow	6	-	-	-	-
Radiology resident	13	-	-	-	-
**Sector**					
Public	21	3	3	2	1
Private	-	0	1	6	-
Both	-	1	8	3	5

HOD, head of department.

† , Less than 5 years of experience post-qualifying;

‡ , 5–15 years of experience post-qualification;

§ , more than 15 years of experience post-qualification. Dash indicates this category is not applicable.

The participants were purposively selected because they worked in the catheterisation laboratory and offered insights into radiation protection issues in this workplace. They were interviewed at conferences or at a suitable location, usually their workplaces, based on appointments scheduled. Snowball sampling was employed and participants were asked to recommend interventionalists they thought would be willing to participate in the study and these were followed up.^23^ We also targeted specific informants, such as the heads of departments, to participate in the study.^24^

### Data collection

We conducted six group interviews of between two and six people and 30 in-depth interviews with individuals. Data collection started in May 2015 and ceased in July 2016 when we determined that data saturation had been reached. Data were collected at four conferences and three workshops in South Africa. These included one Paediatric Interventional Cardiology workshop and two Interventional Radiology workshops. The conferences included two annual conferences for the Cardiology Society of South Africa, one annual conference for the Radiological Society of South Africa and two additional radiology conferences aimed at special interest groups for radiologists. Individuals at radiology, cardiology and paediatric cardiology departments at the university training centres were contacted directly. Selected interventionalists in the private sector were also contacted directly.

A semi-structured interview schedule was used. The questions were open-ended which prompted participants to express their perceptions on the effects of radiation on health, what a culture of radiation safety meant and whether it existed in their workplace, their utilisation of PPE and their thoughts on radiation safety and training and how this could be improved for their respective specialties. Probing questions were asked to get a deeper understanding of participants’ responses.

### Analysis

Thematic analysis using a deductive and inductive approach was used.^25,26^ This analysis was chosen because we wanted to describe and interpret the depth of the experiences of the participants.^27^ We decided on some themes based on the literature (deductive approach) and allowed other codes and themes to emerge from the content of the data (inductive approach). The interviews were audio-recorded and transcribed verbatim. The transcripts were checked against audio recordings for accuracy. Data included the researcher’s field notes.

The data were analysed on an ongoing basis, which included the following steps^27^: Two researchers (André Rose and Kerry E. Uebel) independently read the transcripts and coded the data. We developed a code book as we analysed the data. The codes were organised into categories and the categories were grouped into themes. The interpretations were discussed as they emerged, and the researchers reflected on the disparities and relationships and debated the themes that arose and then reached consensus on the findings. This was an iterative process and the researchers met several times to discuss codes and categories as they emerged.

### Context of the study

Radiologists receive 4 years of training which includes radiobiology and medical physics and it is prescribed in their formal curriculum. They are examined on it as part of their first qualifying examination (usually within their first 18 months of study). It is usually not re-examined in their exit examination.^28^ There is inconsistent coverage of radiation safety in their CME seminars.

Cardiologists have a 3-year training programme to subspecialise in the discipline following qualification as physicians or paediatricians. Radiation safety is mentioned in the syllabus of cardiologists as a learning objective, but it is not formally taught and there is usually poor coverage of it in CME seminars.^28^

South Africa has a two-tiered health system, with an ailing public health system and a thriving and well-established private health system. Interventional radiology and cardiology services are offered in both sectors. It is not uncommon that doctors work in both sectors. Both sectors have challenges, but shortage of equipment such as PPE is common in the public sector. The public sector often has a high patient load with a poor staff complement which does not match the workload.

### Trustworthiness

Our findings are congruent with what is truly happening in practice, based on the researcher’s observations, the data obtained and what is reported in the literature.^29^ The researcher is a (medical) doctor, has worked in a catheterisation laboratory previously and has insight into this work environment. He also spent time observing interventionalists in their workplace.^29^

We conducted in-depth interviews and group interviews which are acceptable and well-described techniques widely reported in the literature.^30^ We achieved site triangulation by including a variety of different interventionalists from different hospitals, cities and from both public and private sectors.^29^ The participants were also at different levels of expertise and included specialists in training to senior specialists and heads of departments. This allowed us to get a better understanding of how the different views converge on an understanding of radiation safety culture in the workplace.^31^

This study has good representation of different categories of interventionalists from across South Africa and offers important qualitative insights into how interventionalists understand what a CRP is. The findings, although not generalisable, can be transferred to the broader South African interventionalist community and similar contexts elsewhere.^29,32^

### Limitations

The qualitative design of the study allowed us to generate hypotheses but not to test them. The study did not investigate the reasons of differences between the cardiologists’ and radiologists’ knowledge of radiation. It also did not fully explore the complexity of what constitutes a CRP and the factors facilitating the creation and establishment of a successful CRP. The sampling strategy restricted the participants in the study and as such offers only the perspectives of the interventionalists and not that of all members of the catheterisation laboratory team. Future studies would add value by exploring these areas and including other members of the catheterisation laboratory team, such as nurses and medical radiation technologists (radiographers).

## Ethical consideration

The study was approved by the Human Research Ethics Committee of the Faculty of Health Sciences of the University of the Free State (ECUFS 44/2015). Written informed consent was obtained from all participants. In the group interviews, the participants were asked not to divulge the responses outside the group. The recordings and transcribed data were stored in a secure place.

## Findings

In this section, we report on the following themes that were identified in the data: ‘culture of radiation protection’, ‘knowledge and awareness of radiation’, ‘radiation safety practice’, ‘PPE utilisation’, ‘monitoring of safety practices’ and ‘education and training’. CRP emerged as an overall theme that intersects with the other themes.

The following notations are used in the article to denote the participants:

- HOD for head of department
- R for radiologists,
- AC for adult cardiologists and
- PC for paediatric cardiologists.
- Cardiologists may refer to either adult or paediatric cardiologists.
- We use the term ‘resident’ to refer to a doctor who is in the process of specialising. In South Africa, residents are referred to as registrars.

### Culture of radiation protection

Culture of radiation protection is a complex overarching theme. It has many facets that intersect with several of the other themes. Participants offered a plethora of perspectives on what constituted a CRP, on how to establish it and how to sustain it. Participants mentioned that it depended on the education and training they received, the role of various stakeholders, monitoring structures, policies and how they were enforced and the dynamics between team members in the catheterisation laboratory.

Radiologists generally expressed the view that they were well informed about radiation safety and felt that they had a well-established CRP within their work environments:

‘But in Radiology Departments it [CRP] is engrained in us. The problem is the rest [other disciplines using ionizing radiation]; it’s not engrained in them.’ [R, HOD]

‘I think we’re more aware than many others. When you take an orthopaedic surgeon or urologist who is using ionising radiation they certainly are not nearly as aware as we are. So, the culture of radiation safety would, I think, depend on all workers, everyone involved in the procedure, everyone in the room all working together to reduce radiation exposure.’ [R]

Cardiologists expressed heterogeneous opinions on the CRP in their workplaces. Some cardiologists stated there was very little attention given to the issue, while others stated they recognised the importance of it, but it was not diligently enforced in the workplace:

‘Radiation control is strictly controlled by guidelines and the problem is we ignore [these] guidelines.’ [R]

The consensus amongst most interventionalists was that the responsibility of creating a CRP lies with several stakeholders. These stakeholders included scrub nurses, radiographers and doctors working in the catheterisation laboratory. There was a general opinion that a top down approach was necessary and that the head interventionalist had to take the lead to establish, maintain and enforce a CRP within the work environment. This view is reflected in the understanding of a radiology resident as follows:

‘It’s about enforcing, and it needs to be top down though, I do agree about that. Your HOD needs to drive it, your consultants need to set good examples and then your registrars who join the program will automatically follow it. No exceptions.’ [R]

### Knowledge and awareness of radiation

There was generally a distinct difference between the knowledge and awareness of cardiologists and that of radiologists pertaining to the effects of radiation. Cardiologists were not unaware that ionising radiation was a hazard, whereas radiologists seemed to engage more easily on how radiation could affect their health and that of their patients. Although the knowledge of these two groups was not quantified, the radiologists appeared to be more knowledgeable:

‘There are two labs, the catheterisation laboratory and the electrophysiology lab and I don’t know the difference in radiation exposure between the two. It would be nice to know the difference. It is a gap in my education.’ [AC]

Many participants commented that because radiation was invisible and the health effects were often long term, proper vigilance was lacking. This resulted in poor radiation safety practices, which potentially compromised their health and that of their patients. The knowledge and awareness about the effects of radiation appeared to be linked with formal training. One cardiologist reflected on awareness of the dangers of radiation only after having had bilateral cataracts removed. There were participants who reflected that they knew colleagues who had experienced negative effects possibly attributed to radiation such as brain tumours:

‘No, I am much more aware after I got the cataracts, I actually had to look at [the situation]. Because I thought why am I getting cataracts and then I thought and said whoa, it must be radiation you know. So, I actually look at radiation now with different eyes.’ [AC]

### Radiation safety practice

Participants were generally not familiar with the policies and legislation that regulate radiation safety. It was of particular concern that heads of department (especially cardiologists) were not well acquainted with the necessary policies, legislation and regulatory bodies:

‘I am not even aware of that [radiation regulations]. So, I don’t know who draws up these guidelines. I don’t know where they’ve been published. Uhm, so if there are any guidelines then it needs to be distributed to the user. It’s pointless drawing up guidelines without uhm, you know informing people, making people aware that these guidelines exist. I am a professor of cardiology and I am not sure where these guidelines are.’ [AC, HOD]

‘So there probably are some policies and regulations [but] we don’t know what they are.’ [PC, Senior]

Participants reflected that radiation safety practice hinged on applying basic principles such as justification for a procedure, the distance maintained from the patient during operation and the X-ray beam, and utilisation of PPE.

Strong opinions were expressed that the practice of radiation safety in the catheterisation laboratory was the responsibility of the individual as well as the entire team. They noted that while individuals were responsible to ensure they had applied principles such as the inverse square law, the ALARA rule and the use of PPE, there also needed to be senior team leaders responsible to check that all staff members complied with standard practice. They generally agreed that the team should have the freedom to challenge other members within the team about radiation safety practices, but recognised that this was complicated by the power dynamic that existed between differently ranked professionals within the team. This power disparity was particularly evident in the private sector where participants reported that the rest of the team was unlikely to challenge the doctor. It appeared that the public sector doctors were willing to abide by structures put in place to ensure their safety and were more willing to be instructed by, for example, a scrub nurse or radiographer. The attitudes of some doctors and an unwillingness to listen to others in the team were noted as barriers to good radiation safety practice:

‘And part of their bad habits is their egos because they don’t want to be told by the radiographer take your foot off the pedal. “I’m in charge, I am the big doctor here.”’ [R, HOD]

Participants expressed the view that radiation safety practice was also the responsibility of stakeholders that did not ordinarily form part of the catheterisation laboratory team. These actors included the radiation safety officers, the medical physics department and the hospital management. Participants also mentioned the critical role of hospital management in ensuring adequate quality control, maintenance and timely replacement of machines and PPE:

‘The HOD and CEO need to be responsible for ensuring equipment works and is fixed.’ [R]

‘Hospital management needs to ensure equipment is kept up to date and maintained properly.’ [AC]

There was poor awareness about the existence, role and function of radiation safety officers. Participants were not always sure what role the medical physics department played or should play. In some instances, they were not even aware of the existence and function of such a department within their facilities:

‘I don’t know the name of the radiation control board.’ [AC]

‘There is no communication between us and the radiation control board.’ [AC]

A dominant view that emerged from the study was that the participants felt a strong sense of responsibility to their patients, even if performing a procedure without adequate radiation protection would jeopardise their own safety and health. This attitude underpins the culture of care that exists with some doctors. Procedures were sometimes performed self-sacrificially in the interest of the patient, but at the cost of compliance with standardised radiation safety practices:

‘[Be]cause our job is about the patient and that’s why we do what we do. And that’s actually how the whole of medicine generally works. […] So, it’s simply not in our makeup [to think about ourselves]. […], but you know ninety-nine percent of our focus is for the patient and then the other one percent is for maybe a few other things.’ [PC]

### Personal protective equipment utilisation

Participants noted both barriers and facilitators to PPE utilisation. Barriers included poor availability and quality control, poor fit, inadequate size ranges and equipment being cumbersome to use. Factors that facilitated the use of PPE included the hospital management accepting responsibility to supply PPE and individual interventionalists also taking responsibility to use PPE and to purchase their own PPE in cases where it is not readily available in their facility.

Personal protective equipment utilisation was not quantified in this study, but the most commonly reported PPE used were lead aprons. Thyroid guards were less commonly used. Lead glasses were either used very infrequently or not at all. Ceiling suspended shields were hardly ever used, but were frequently reported not to be available. There was generally very poor awareness of ceiling suspended shields and mobile shields. Only one participant indicated that he used lead gloves and a lead cap.

Participants reported that PPE was not always available. They usually had sufficient lead aprons, but other PPE was frequently unavailable in the public sector, with a notable shortage or absence of lead glasses and thyroid shields. Lead glasses were particularly in short supply and were often not replaced when damaged or lost:

‘It becomes such a hassle to try and get a thyroid shield and to try and get goggles that you just don’t. You don’t have enough time in a day to try and look for it [be]cause you never gonna find it in any case. So, then you just ignore it and you go with the lead apron only. That’s what most of us do.’ [R, resident]

Participants who worked mainly in the public sector also noted the lack of availability of different sizes and designs of PPE as a problem. They noted that they did not have access to newer more ergonomically designed PPE and were often forced to settle for equipment that was too heavy or ill-fitting. The participants generally held the opinion that they wanted a range of choices for PPE. Women participants stated that some equipment was inappropriate for their physique. A female radiologist made the following remark:

‘The problem is the size, [be]cause we have one pair or two pairs. There’s […] one size for everyone, we [are] not all the same sizes so for some of us it may not fit. [R]

The ability to perform procedures dexterously strongly influenced PPE utilisation. Many participants described that lead glasses were awkward to use during procedures and cannot be used over prescription glasses. One participant stated:

‘They [lead glasses] are bulky, they mist up and they cause pressure on the nose.’ [AC]

‘[…] the challenges are if you using prescription glasses. Because then you have to make yourself a set of lead prescription glasses. It’s a bit expensive, but if you realise the environment that you [are] working in uhm, [then] you could invest bit of money towards your own safety. […]. Cardiologists are making a lot of money, but a blind cardiologist doesn’t mean anything.’ [R]

There was consensus amongst participants that hospital management was responsible for supplying adequate PPE for their staff and for ensuring adequate and continuous quality control of PPE:

‘So, I would imagine it’s the hospital’s responsibility [to provide PPE]. I mean we’re employed by the hospital. The hospital has a responsibility to all its employees to maintain [their] safety.’ [PC]

‘It’s the hospital’s responsibility to provide that [PPE]. It’s my responsibility to use it.’ [R]

Many participants also reflected that individuals had a responsibility to use PPE regularly and that owning their own personal PPE could act as a stimulus for consistent utilisation:

‘Hmm, it becomes second nature, I mean it comes with the turf, hmm, it’s almost like my uniform I mean you don’t even think about having to put on your lead apron when you [are] going to do a procedure, to put on your thyroid shield, your collar. I mean those things come almost automatic to you now.’ [AC]

### Monitoring of safety practices

Participants raised the issue of monitoring of radiation safety and stated there was inconsistent use of dosimeters, lack of feedback on the radiation they were exposed to and there was a need for more regular occupational medical examinations.

Many participants reported inconsistent utilisation of personal dosimetry badges either because they forgot to wear their dosimeters, they were not held accountable to wear them, they received no feedback about previous readings, or worryingly because high readings might limit the work they could do in the catheterisation laboratory:

‘No, I must tell you honestly sometimes I am working so hard that’s [radiation safety] really the last thing on my mind. You always wear protective gear, but I can honestly tell you I don’t wear my radiation badge at all, never. It’s in my bag and that’s it. I don’t wear it when I’m doing screening, I don’t wear it when I’m doing angiography and I don’t wear it, no.’ [R, resident]

‘We don’t get regular reports of our radiation doses.’ [PC]

Most participants commented that they did not receive sufficient regular feedback on doses they were exposed to. Many were unaware of the medical physics departments at their facilities or what role these departments should be playing in monitoring their dose exposure. This lack of feedback on their dosimetry readings not only influenced their inconsistent use of dosimeters but also denied them of the opportunity to improve their radiation safety practices by being able to check monthly doses received:

‘You wear dosimeters and it seems as if nobody checks them or does anything.’ [AC]

Participants commented that real-time monitoring would improve their radiation safety practice, as it would give an immediate indication of their own and the patients’ radiation exposure and aid to plan future procedures.

Some participants reflected that they almost never received regular periodic occupational health examinations. This was especially true for the private sector.

### Education and training

We identified two broad categories with respect to education and training. Firstly, formal education and CME, which interventionalists receive as part of their training. Radiologists received formal instruction in radiobiology and radiation physics, which was part of their core curriculum, while cardiologists received little or no formal instruction in it. Radiologists were generally satisfied with the level of formal education they received in the discipline but noted that they thought that other medical disciplines using ionising radiation such as cardiology, orthopaedics and urology were undertrained in the subject. A head of the department (HOD) in radiology reflected on the discrepancy in training between different medical disciplines using radiation as follows:

‘The interventional group and people that I work with, there are no bad habits in that group. The bad habits are with our friends the cardiology group, orthopods and vascular surgeons. Because the vascular surgeons that are doing interventional work are not being trained as a radiation worker so they’ve got bad habits.’ [R, HOD]

‘Many other disciplines do not have the training in radiation that radiologists have.’ [R]

The cardiologists expressed differing views on their formal training ranging from not deeming it necessary to receive formal instruction to expressing a definite need to have such instruction. A senior paediatric cardiologist stated, ‘We can add some training [on radiation safety] because at the moment there isn’t any’. In contrast, an HOD in cardiology expressed the view that there was no need to formally train cardiologists in radiation safety.

Many of the cardiologists and radiologists described the need for proper induction and that radiation safety should be comprehensively covered in CME activities and at conferences. A radiologist from New Zealand reflected that work induction in the radiation workplace was lacking and is needed:

‘Ah, I think it [induction] often gets forgotten, I think the nurses are orientated, ah, I think it is probably needed; we need a more formal process. And I think in particular the doctors, they go into the lab and it is assumed that the doctors understand about radiation [but] that isn’t the case.’ [R]

Secondly, the issue of mentorship by more senior specialists was a way for fostering a culture of education and radiation safety training. Junior participants expressed the view that it was the responsibility of senior specialists to provide guidance on education and training in radiation safety:

‘Well, it’s not senior people’s responsibility [to get junior doctors to wear PPE], but junior doctors often will take the lead if the more senior doctors do so, if you are a junior interventionalist and your mentor doesn’t wear that [PPE] you’re less likely to wear them.’ [AC]

We have included the views of three international interventionalists who were interviewed at the conferences because they corroborate the views of the South African interventionalists. The experiences of the international interventionalists and their South African counterparts were similar.

## Discussion

In this section, we offer a synthesis of what constitutes a CRP based on the themes that emerged. We explore organisational and workplace culture, safety culture and how training and education fosters development of a CRP.

### Organisational and workplace culture

Organisational culture in a health facility is a complicated construct that is built on the values, attitudes and norms within an organisation.^10^ It is these beliefs, norms, attitudes and values that create the governance framework that prescribes acceptable and expected behaviour within an organisation.^33^ The behaviour evolves because of the social constructs of the employees, their professional background and its underpinning ethos, the rules and regulations that govern their workplace and the influence of managerial and administrative structures.^18,19^ Organisational behaviour shapes the culture within an organisation and determines the success of the health facility.^34^

The behaviour in the organisation is moulded at an individual and group level.^35^ The catheterisation laboratory is a highly contextualised work environment. In this workplace, doctors often leverage a power dynamic over other members of the team. There may also be a gender disparity between team members as the interventionalists are still mainly males and the nurses and radiographers (radiology technicians) are predominately females. This may hinder members of the team such as nurses and radiographers from challenging the non-compliant safety practices of interventionalists. This interdisciplinary power dynamic and gender disparity are characteristics of many workplaces (catheterisation laboratories) in South Africa.

In our findings, participants highlighted this difference, illustrating that other members of the catheterisation laboratory team like nurses and radiographers may feel like they could not challenge doctors. Interventionalists stated that this power imbalance may be a barrier to fostering a CRP in the workplace. Changing this behaviour is difficult and poses a hurdle to establishing a healthy and inclusive responsibility in developing a CRP. However, culture is a dynamic construct and malleable to change.^19^ It is this strength that should be leveraged to challenge and change the prevailing culture within the catheterisation laboratory.

Change management principles can help disrupt this hierarchal behaviour. There are several change management strategies that can be used to bring about this change. The steps in the change process proposed by Kotter’s model underpin several of these change strategies, making it an ideal change management strategy.^36,37^ These steps include: (1) creating urgency for the change process, (2) forming the partnerships needed for the change, (3) formulating a vision and strategy, (4) communicating the change vision, (5) empowering the key stakeholders for the change and action required, (6) establishing short-term goals, (7) consolidating the change and facilitating further change and (8) cementing these attitudes within the culture.^36,37^

Negotiating this change involves communicating the message that every member of the catheterisation laboratory team has a responsibility to their personal safety, the team’s safety and the patient’s safety. Radiation safety is a collective effort. The change needed to create a CRP depends on everyone’s participation and not solely on an individual.^35^

A CRP hinges on a symbiotic relationship between the catheterisation laboratory team and the facility’s managerial team. How this relationship forges itself is indicative of the resulting CRP. The relationship between healthcare managers and clinical staff is often contentious because of competing priorities. Healthcare managers often prioritise cost-saving measures, which may compete with safety measures.^38^ Health budgets, profit margins and cost-saving measures are an important consideration for health managers, which may shape decisions about procurement of new equipment, maintenance of existing machines and the acquisition of PPE. These objectives can be a barrier to a culture of safety. It is thus important that the head of the catheterisation laboratory must be astute in negotiating resources for safety. This is especially pertinent in resource-constrained environments. The South African regulations stipulate what type of PPE must be worn and this can also be leveraged when negotiating for resources.

Different values compete to create one of the following four organisational cultures (or a combination of them): clan culture (cohesive, participatory leadership), developmental culture (creative, adaptive leadership), hierarchical culture (leadership bound by rules and policies) and rational culture (competitive and goal orientated).^33^ Participants suggested that a hierarchical structure should be fostered. This may, however, hinder rather than facilitate a CRP. A hierarchal structure favours tension between doctors and other professionals and between managerial structures and clinicians.^19^ A participatory and inclusive approach would be an enabling approach in the co-creation of a CRP between the various team members.^39^

### Safety culture

A culture of safety is imperative in any workplace. The attitude towards safety is part of the underpinning values of an organisation and the cornerstone for developing good radiation safety practices. A safety culture is cultivated by the response of HCWs to PPE utilisation and the monitoring structures that keep these practices in check.

Safety and care should interact synergistically with each other. A culture of care is a balance between the science and humanity of medicine.^40^ Doctors (and other HCWs) wrestle with the challenge of balancing patient-centred care (PCC) with sophisticated technologies and the necessary safety precautions that accompany it. Participants indicated that they would rather perform interventional procedures without PPE than compromise the care of patients. This action, though seemingly noble, is a breach of safety practices and places the HCW and the patient at risk and in so doing compromises the safety and quality of care. PCC has to be holistic, placing the patients’ safety and the quality of care at its centre and drawing on collaboration of all members of the health team.^41^

The occupational health risks associated with ionising radiation may be offset with consistent and appropriate PPE utilisation.^42^ Consistent use depends on the availability and fit of the PPE, education in using it correctly and the dexterity to perform procedures with it in place.^15,43^ Interventionalists reported attitudes and behaviour to PPE use congruent with findings in the literature and compliance depended on availability and proper fit.

There are personal and systemic factors that influence the use of PPE. The individual primarily makes the decision to use the PPE, but this behaviour may be influenced by the prevailing safety culture within the organisation. A strong safety culture will positively affect the use of PPE.^44^ It is thus crucial that the culture within the catheterisation laboratory embraces and encourages such a culture as a norm.

Participants expressed the view that managerial structures were responsible for ensuring provision of PPE. Managerial structures are an important enabler to ensure availability of PPE and to foster a CRP.^8^ Together with the person designated to oversee radiation safety in the catheterisation laboratory and the radiation officer, they can ensure that appropriately fitting PPE is procured timely for the catheterisation laboratory team. Provision of PPE facilitates increased uptake of utilisation of PPE.^16^

Dosimeter monitoring is an important component of regulating radiation exposure in the workplace.^7^ The monitoring offers the users an opportunity to know the radiation exposure they are receiving and to adjust operating procedures’ exposure time to reduce the dose exposure to themselves and the patients.^8^ Interventionalists are notorious for poor compliance and inconsistently using their dosimeter badges.^45^ Developments in individual dosimetry monitoring such as real-time and wireless monitoring systems will promote better compliance.^2^ A CRP would help encourage a different attitude towards using dosimeters and facilitate radiation monitoring in the workplace.

Incorporation of an occupational health surveillance system would be integral to radiation safety monitoring. The unique nature of occupational radiation exposure would require a bespoke medical system for this category of work.^46^

### Education and training

Interventionalists need to be educated and trained so that they can understand the risks and benefits of using ionising radiation.^47^ Education and training helps stimulate and sustain a CRP. However, training should be regular and consistent to ensure reinforcement and continued adherence.^12^ Training aids in instilling autonomy towards radiation safety in radiation operators.^48^ The training curriculum on radiation safety is inadequate for South African cardiologists and the gap in this training requires urgent attention.^49,50^ The training needs to be included in the formal training curriculum and in the CME programme to ensure consistency in practice.^12^ Training provokes improvement in the radiation management skills of operators, which improves the safety of all team members and patients in the catheterisation laboratory.^51^ The theme of education and training related to this study is extensively explored elsewhere.^49^

The results from this study are significant because they provide insights into the work and organisational culture in this highly contextualised work environment. They highlight risky occupational practices that can be mitigated with change management strategies. Africa is acquiring high-tech imaging equipment at an accelerated rate, but the workplace culture that should match this change is lagging and poses a potential health risk to a highly skilled workforce and the patients they serve. Furthermore, the study is significant because it offers insights for establishing policies and practice guidelines for developing a CRP in this work environment. These insights are universally applicable.

## Conclusion and implications for practice

The increased utilisation of ionising radiation in modern medicine is likely to remain unabated, but the escalation in the number of procedures performed and the increase in the complexity of the procedures will result in operators being exposed to larger cumulative radiation doses. Mitigating this occupational risk will require greater vigilance in promoting a CRP. Developing and strengthening this culture requires a change in organisational and workplace culture towards radiation safety. A well-developed and continuous education programme is imperative to initiating and sustaining a CRP. A well-developed CRP promotes patient safety and improves quality of care. It requires commitment from both the radiology and cardiology fraternities to embrace a paradigm shift towards garnering support for radiation safety in the workplace.
